# Preliminary evidence that blocking the uptake of placenta-derived preeclamptic extracellular vesicles protects the vascular endothelium and prevents vasoconstriction

**DOI:** 10.1038/s41598-023-45830-9

**Published:** 2023-10-27

**Authors:** Lena Erlandsson, Lena Ohlsson, Zahra Masoumi, Mimmi Rehnström, Tina Cronqvist, Lars Edvinsson, Stefan R. Hansson

**Affiliations:** 1 Division of Obstetrics and Gynecology, Department of Clinical Sciences Lund, Lund University, BMC C14, Klinikgatan 28, 221 85 Lund, Sweden; 2 Experimental Vascular Research, Department of Clinical Sciences Lund, Lund University, Lund, Sweden; 3 Skåne University Hospital, Lund, Sweden

**Keywords:** Pre-eclampsia, Experimental models of disease, Molecular medicine

## Abstract

Preeclampsia (PE) is a pregnancy syndrome characterized by hypertension and organ damage manifesting after 20 gestational weeks. The etiology is of multifactorial origin, where placental stress causes increased levels of placenta-derived extracellular vesicles (STBEVs) in the maternal circulation, shown to cause inflammation, endothelial activation, vasoconstriction, and anti-angiogenic activity. General endothelial dysfunction is believed to be initiated by endothelial insult during pregnancy that alters vascular function resulting in increased arterial stiffness, cardiac dysfunction, and increased risk of cardiovascular disease later in life. We compared the effect of normal and PE derived STBEVs in vitro on vascular contractility of human subcutaneous arteries using wire myography. Cellular structures of exposed vessels were investigated by transmission electron microscopy. We explored strategies to pharmacologically block the effects of the STBEVs on human vessels. The PE STBEVs caused significantly stronger angiotensin II-mediated contractions and extended structural damage to human subcutaneous arteries compared to normal STBEVs. These negative effects could be reduced by blocking vesicle uptake by endothelial cells, using chlorpromazine or specific antibodies towards the LOX-1 receptor. The therapeutic potential of blocking vesicle uptake should be further explored, to reduce the permanent damage caused on the vasculature during PE pregnancy to prevent future cardiovascular risk.

## Introduction

Preeclampsia (PE) is a complex pregnancy syndrome that is characterized by hypertension and organ damage manifesting after 20 weeks of gestation. The etiology is believed to be of multifactorial origin and outlined in the two-stage model for PE^1^ where poor placentation, due to shallow trophoblast invasion, is followed by syncytiotrophoblast stress caused by placental malperfusion and oxidative stress. These events are thought to be associated with early-onset PE (delivery < 34 gestational weeks (GW)), affecting around 20% of PE cases. Late-onset PE (delivery ≥ 34 GW) is more associated with maternal risk factors where syncytiotrophoblast stress is induced by maternal constitutional factors and/or environmental factors such as air pollution^2^. In both scenarios, placental cellular stress causes the release of abnormal placenta-derived material into the maternal circulation where it induces an inflammatory response and endothelial damage^3^. This leads to stage two, the clinical manifestations characterized by hypertension, proteinuria and/or end-organ dysfunction caused by increased systemic vascular resistance and endothelial dysfunction. In normal pregnancies vascular adaptations such as reduced systemic vascular resistance and increased resistance to angiotensin II (Ang II) facilitate maintaining normal blood pressure^4^. In contrast, pregnancy complications such as PE fail to reach these hemodynamic adaptations^5^.

General endothelial dysfunction and injury due to persistent endothelial damage is well established in the pathophysiology and organ failure seen in PE pregnancies^6^. Women with PE are described to have increased arterial stiffness^7^, cardiac dysfunction^8^, and an increased risk of cardiovascular disease (CVD) later in life^9^. The permanent endothelial damage following PE can be described as the third stage of the disease, and is believed to be initiated by the endothelial insult during pregnancy that alters vascular function at the genomic level by epigenetic modifications^10^. There have also been suggestions of underlying endothelial dysfunction prior to pregnancy, causing placenta-related defects such as PE and recurrent pregnancy loss^11^ as well as known risk factors involving endothelial damage such as diabetes. However, the underlying molecular mechanisms need to be further deciphered.

The placenta normally releases syncytiotrophoblast-derived extracellular vesicles (STBEVs) into the maternal circulation, that interact with maternal cells and regulate maternal response to pregnancy. The STBEVs carry a variety of biologically active molecules, such as lipids, proteins, or nucleic acids^12,13^ and are taken up by endothelial cells through clathrin-mediated endocytosis, which can be completely blocked by the pharmaceutical compound chlorpromazine^14^. However, the higher levels of STBEVs in plasma in PE ^15,16^ and their altered cargo^17^ may contribute to the altered nitric oxide (NO) bioavailability, vasoconstriction, endothelial activation and inflammation seen in PE.

Angiotensin II signaling is part of the renal and cardiovascular physiology, and an important regulator of blood pressure mediated by the renin-angiotensin pathway^18^. Interestingly, PE women have significantly increased vascular sensitivity to Ang II, and reduced endothelium- and NO-dependent dilation^19^, which can be reversed by pharmacological block of the Ang II type I receptor (AT1R). The AT1R forms a complex in the cell membrane with the lectin-like oxidized LDL receptor-1 (LOX-1) thereby interconnecting their signaling pathways^20^. The LOX-1 is a multi-ligand scavenger receptor, binding oxidized low-density lipoproteins (oxLDL), mainly expressed on endothelial cells at low levels under physiological conditions. It is upregulated in pathophysiological conditions such as PE^21^ and reported to be involved in vascular dysfunction in several cardiovascular diseases^22,23^. The connection between the receptors mediates oxLDL-induced activation of AT1R, resulting in internalization of the oxLDL-LOX-1-AT1R complex via a β-arrestin pathway involving clathrin-mediated endocytosis^24^. Once inside the cell, oxLDL was found to co-localize with early and late endosomes. In vitro experiments have shown that STBEVs can bind to and activate LOX-1, mediating effects on endothelial function and NO availability which could be inhibited by an anti-LOX-1 blocking antibody^25^.

Endothelin-1 (ET-1) is a potent endothelium-derived vasoconstrictor that is elevated in different cardiovascular diseases in humans, and elicits its effects via two receptors, ET_A_ and ET_B_^26^. The ET_A_ receptor is mostly expressed on vascular smooth muscle cells (SMC), while the ET_B_ receptor is expressed on vascular endothelial cells as well as SMCs^27^. Internalization of ET-1 and its receptor occurs mainly via caveolae instead of clathrin-coated pits^28^. Circulatory ET-1 levels are elevated in PE and thought to play a role in its pathophysiology^29^. In addition, Ang II stimulates the expression of ET-1 in vascular endothelium^30^ and AT1R-agonistic autoantibodies produced during PE increase the secretion of ET-1 from vascular endothelium through activation of AT1R^31^.

In this study, we compared the effects of human STBEVs isolated from ex vivo perfused normal and PE placentas^32^ on vascular contractility of human subcutaneous arteries from normal pregnancies, using in vitro wire myography. Cellular structures affected by exposure to STBEVs were investigated by transmission electron microscopy (TEM), while the STBEVs interaction with the vessel wall was visualized by immunoelectron microscopy. Lastly, we explored two different strategies to pharmacologically block the uptake of vesicles to prevent their vascular effects.

## Methods

### Ethics statement

The collection of STBEVs from normal and PE placentas by dual ex-vivo perfusion of human placental cotyledons has been described previously^33^, and was approved by the Oxfordshire Research Ethics Committee C at Oxford University, UK. Informed consent was obtained from all participants and clinical data was collected (Table 1). For the isolation of human subcutaneous arteries from healthy women undergoing Caesarean section after a normal pregnancy, the study was approved by the Ethics Committee Review Board for studies on human subjects at Lund University and Skåne University Hospital, Lund, Sweden (Dnr LU-818–01). Informed consent was obtained from all participants. Women contributing with subcutaneous biopsies were normotensive pregnancies without complications or any known risk factors for PE, and with equal distribution between fetal sex. Elective Caesarean section was performed due to indications such as breech or psychosocial reasons (Table 2). All experiments involving human tissue biopsies were performed according to the Helsinki Declaration.

**Table 1:** Patient demographics for placentas from singleton pregnancies used for placenta perfusion to collect STBEVs. Data is presented as median values (min–max) for each group.

	Normal (n = 13)	PE pregnancies		
		PE total (n = 12)^a^	EOPE (n = 4)	LOPE (n = 8)
Maternal age (years)	36 (31–41)	31.5 (23–44)	29.5 (28–38)	32 (23–44)
GA (weeks + days)	39 + 1 (38 + 1–41 + 4)	35 + 1 **** (27 + 1–39 + 2)	28 + 4 ***/**^b^ (27 + 1–30 + 0)	36 + 2 *** (34 + 0–39 + 2)
Max sys BP (mmHg)	125 (90–156)	167 **** (142–200)	179 **/– (150–195)	162 **** (142–200)
Max dia BP (mmHg)	75 (50–92)	103 **** (92–122)	116 ***/* (110–122)	100 **** (92–121)
Fetal weight (g)	3990 (3110–4375)	2079 **** (760–3500)	960 ***/** (760–1200)	2352 *** (1710–3500)

^a^The PE group is a mix of EOPE (n = 4) and LOPE (n = 8). BP = blood pressure; dia = diastolic; EOPE = early-onset PE; GA = gestational age; LOPE = late-onset PE; PE = preeclampsia; sys = systolic. Mann–Whitney test, comparing Normal with PE, EOPE or LOPE, and ^b^EOPE with LOPE: *p < 0.05, **p < 0.01, ***p < 0.001, ****p < 0.0001.

**Table 2:** Patient demographics for normal singleton pregnancies used for the isolation of human subcutaneous arteries. All were delivered by Cesarean section due to indications such as breech or psychosocial reasons. Data presented as median value (min–max).

	Normal (n = 20)
Age (years)	36 (29–44)
Gestational age (weeks + days)	38 + 4 (36 + 3–39 + 1)
Parity	1 (0–3)
BMI^a^	22 (17–33)
Fetal weight (g)	3477 (2393–4378)

^a^A calculated BMI was registered in the clinical data for 13 out of 20 patients. For the rest (7 of 20) BMI was only registered as normal (< 25).

### Placental STBEV preparation

Normal and PE STBEVs were isolated from stored maternal perfusate media, collected at dual ex-vivo perfusion experiments performed on placentas from normal (n = 13) and PE (n = 12) pregnancies (Table 1). These perfusates were used in previous studies^14,32,34^, but the remaining amounts were too small to be used as individual samples, or to be used as pooled early-onset and late-onset PE, respectively. All isolated PE STBEV’s were therefore pooled (n = 12; a mix of early-onset (n = 4) and late-onset PE (n = 8)), as were the normal STBEVs (n = 13). Early-onset PE was defined as < 34 gestational weeks, and late-onset PE as ≥ 34 weeks. Briefly, as described previously, the placenta perfusion experiment began with an equilibration phase of 30 min, from which the perfusate was discarded. After equilibration, the maternal circuit was closed, and the placenta was perfused for 3 h. Perfusate was collected from the maternal side and used as a source of normal and PE STBEVs. The STBEVs were isolated from the collected perfusion medium by centrifugation as previously described^14^. Briefly, the maternal perfusate was centrifuged at 1500×*g* to remove any cells released from the placenta. The supernatant was centrifuged at 3500×*g* at 4 °C for 20 min to remove cellular debris, and thereafter ultra-centrifuged at 110,000×*g* at 4 °C for 3 h. The pelleted STBEVs were re-suspended in phosphate buffered saline, pooled into normal and PE STBEVs, and thereafter stored at −80 °C until use. The STBEVs collected from these perfusates have previously been characterized^14,32,34^, demonstrating similar protein concentration (using a NanoDrop Spectrophotometer ND-1000 (NanoDrop Technologies, Wilmington, USA)), particle concentration and size distribution (by Nanoparticle Tracking Analysis (NTA) using a NanoSight LM10 (Nanosight Ltd., Amesbury, UK)). Lastly, both normal and PE STBEVs have been shown to be positive for the STBEV surface marker placental alkaline phosphatase (PLAP) (by Western blotting, using primary anti-PLAP antibody NDOG2 (provided by Professors Ian Sargent and Manu Vatish, UK)). Studies have shown that placental perfusion experiments generates high STBEV yields^35^, with low level of contamination from other sources^36^. In addition, isolated STBEVs from placental perfusions are considered resembling physiologically produced STBEVs^37^, with minimal structural damage to the placenta.

### Preparation of human subcutaneous arteries

Subcutaneous arteries for myography and in vitro exposure experiments were harvested from biopsies taken from abdominal subcutaneous fat from healthy women undergoing elective Caesarean section in a normal term pregnancy (n = 20) (Table 2), and immediately put in cold DMEM-medium or sterile saline solution. Small unbranched arteries were dissected under light microscopy and transferred to cold Na-Krebs buffer (119 mM NaCl, 15 mM NaHCO_3_, 4.6 mM KCl, 1.2 mM MgCl_2_, 1.2 mM NaH_2_PO_4_, 1.5 mM CaCl_2_, 5.5 mM glucose, pH at 7.3–7.4), as previously described^38^. For use in myography experiments, vessels were cut into 1–2 mm ring segments within 1 h from collection. The diameter of the segments varied between 193–443 µm, with a mean diameter of 301 ± 22 µm. The dissected vessels were either kept in Na-Krebs buffer overnight at 4 °C or used the same day for myography experiments. For use in in vitro STBEV-exposure experiments, vessels were cut into 3–5 mm lengths within 1 h from collection and used the same day.

### Myograph experiments

Wire myography that records isometric tension^39^ was used to measure vasoconstriction responses in subcutaneous human blood vessels as previously described^38^. Each segment was mounted on two stainless steel wires (40 µm) connected to a device sensitive to vascular tension. The mounted segments were immersed in temperature-controlled (37 °C) tissue baths containing Na-Krebs (continuously aerated with 5% CO_2_ to maintain pH at 7.3–7.4). The arterial stretching force property was recorded with a Mulvany–Halpern myograph (Danish Myo Technology A/S, Aarhus, Denmark). Each segment was stretched to 90% of its normal internal circumference, which is the size each segment would have in a transmural pressure of 100 mm Hg. After normalization, the vessels were washed with fresh warm buffer and allowed to rest for 30 min. The maximum contractile capacity of each arterial segment was assessed twice by exposure to 60 mM KCl buffer (part of the NaCl in the Na-Krebs buffer solution was replaced with 60 mM KCl), with one wash and one rest in between, and the mean set as the 100% level for comparison to substance responses. This viability test also examined stable contractions and gave information on the condition of each individual artery segment. After the second exposure to 60 mM KCl buffer, the vessels were washed and allowed to rest for 10–30 min until a stable baseline was achieved. The endothelial integrity of the vessels was tested with pre-contraction induced by 5-hydroxytryptamine (3 × 10^−7^ M, Sigma Aldrich, Merck Life Science AB, Sweden), followed by a 3–5-min dilation response induced by carbamylcholine chloride (carbachol, 10^–5^ M, Sigma Aldrich, Merck Life Science AB, Sweden). After washing the vessels with Na-Krebs buffer, baseline was again stabilized before the addition of either chlorpromazine (10 µg/ml, Sigma Aldrich, Merck Life Science AB, Sweden), candesartan (AT1R-receptor antagonist, 1 nM, Tocris Bioscience, Bio-Techne Ltd, UK), FR139317 (ET_A_-receptor antagonist, 1 µM, Tocris Bioscience, Bio-Techne Ltd, UK) or buffer, followed by incubation for 30 min. This was followed by the addition of 40 µg/mL PE or normal STBEVs and incubation for 30 min. We have previously shown that placenta-derived STBEVs are internalized by human endothelial cells within 30 min of incubation^32^. Individual receptor-mediated responses were evaluated by cumulative application of each receptor agonist. Firstly, we investigated effects on the Ang II receptors AT1R or AT2R by cumulative administration of Ang II (Sigma Aldrich, Merck Life Science AB, Sweden) at concentrations from 10^−12^ to 10^−8^ M, followed by a wash and a pause until the baseline was restabilized. Thereafter, the effects on the ET-1 receptors ET_A_ and ET_B_ were investigated by cumulative administration of ET-1 (Sigma Aldrich, Merck Life Science AB, Sweden) at concentrations from 10^−11^ to 10^−7^ M. The myograph experiments were recorded by LabChart software (ADInstruments NZ Limited, New Zealand) and results calculated for each vessel segment. Responses were calculated as the percentage of the maximum contraction obtained with 60 mM KCl.

### In vitro STBEV exposure of human subcutaneous arteries

For in vitro STBEV-exposure experiments, prepared vessel segments were immersed in carbonated Na-Krebs buffer (pH 7.3–7.4) in Eppendorf tubes, while gently shaking. Vessels were incubated for 2 h at 37 °C with buffer, or buffer containing 40 µg/ml PE or normal STBEVs with and without 10 µg/ml chlorpromazine (Sigma-Aldrich) or 10 µg/ml anti-LOX-1 antibody (TS92, provided by Professor Tatsuya Sawamura, Japan). The vessels were thereafter fixed for further microscopy analysis. The experiment was run twice. As stated above, we have previously shown that placenta-derived STBEVs are internalized by human endothelial cells within 30 min of incubation^32^.

### Transmission electron microscopy

Following in vitro STBEV-exposure experiments, the vessel segments were transferred to fixative solution (1.5% paraformaldehyde and 1.5% glutaraldehyde in 0.1 M Sorensen’s buffer pH 7.2) and fixed for one hour at room temperature, followed by a wash and overnight incubation at 4 °C in Sorensen’s buffer. Fixed samples were prepared for ultrathin sectioning and subjected to TEM as reviewed in^40^.

Gold-labeled antibodies were used in immunoelectron microscopy localization of STBEVs on sectioned vessels. An antibody directed against PLAP, expressed on placenta-derived extracellular vesicles (NDOG2, provided by Prof. Ian Sargent and Prof. Manu Vatish, UK) was used together with a colloidal gold labelled goat anti-mouse secondary antibody (10 nm, Ted Pella Inc, Redding, California, USA) to detect placenta-derived STBEVs. An anti-LOX-1 antibody (TS92, provided by Prof. Tatsuya Sawamura, Japan) was used together with a colloidal gold labelled goat anti-human secondary antibody (10 nm, Ted Pella Inc, Redding, California, USA) to detect LOX-1 in the vessel wall. Samples were treated with 1% BSA plus 1% goat serum to block unspecific staining. Microscopy analysis was performed on two sections from each vessel segment. Negative control image is shown in supplementary information (Supplementary Figure S4A).

### Statistics

GraphPad Prism software (Version 9.3.1, San Diego, California, USA) was used to create figures and perform statistical analysis. Data is presented as mean ± SEM of the number of observations indicated. A p-value < 0.05 was considered statistically significant. Non-parametric Wilcoxon’s matched-pairs signed-ranks test was used to compare differences in obtained responses to added substances together with different types of STBEVs, and with or without chlorpromazine block.

The maximum contractile response (E_max_ %) for each tested substance was obtained for the vessel segments as percentage of the contractile response to the initial 60 mM KCl. The negative logarithm of the half maximum effective concentration (pEC_50_) was obtained for the purpose of comparing the potency of different substances, calculated using the function log (agonist) vs. response -Variable slope in the GraphPad Prism software (Version 9.3.1, San Diego, California, USA).

## Results

### STBEV characterization

There was no significant difference between normal and PE STBEVs regarding size or particle concentration. The NTA analysis of isolated normal and PE STBEVs before pooling the samples showed similar particle concentrations, with 8.5 × 10^8^ ± 4.6 × 10^7^ and 7.8 × 10^8^ ± 2.5 × 10^7^ particles/ml, respectively. Vesicle size ranged between 50–450 nm (Supplementary Figure S1), with a mean value of 135 ± 15 nm and 141 ± 19 nm for normal and PE STBEVs, respectively. The protein concentration for pooled normal and PE STBEVs were also similar at 1.6 ± 0.6 mg/ml and 2.2 ± 1.0 mg/ml, respectively.

### Vascular contractility

**Figure 1:**
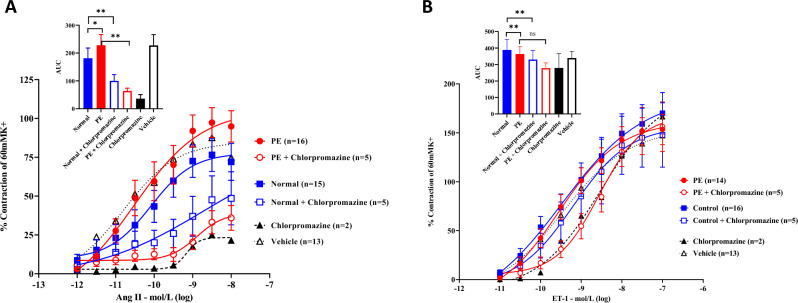
Myograph analyses of STBEV exposed mounted human arterial vessels. Human arterial vessels mounted in a myograph and incubated with normal or PE STBEVs. (**A**) Contractility response to normal or PE STBEVs at cumulative concentrations of Angiotensin II, and with or without the addition of chlorpromazine. In insert, non-parametric Wilcoxon’s matched-pairs signed-ranks test was used to compare differences in responses as AUC. (**B**) Contractility response to normal or PE STBEVs at increasing concentrations of Endothelin-1, and with or without the addition of chlorpromazine. In insert, non-parametric Wilcoxon’s matched-pairs signed-ranks test was used to compare differences in responses as AUC. Data is presented as mean ± SEM for each data point. *p < 0.05, **p < 0.01. AUC = area under curve; PE = preeclampsia; Vehicle = buffer without STBEVs.

Mounted arterial vessel segments were incubated with pooled normal or PE STBEVs, with or without the uptake blocker chlorpromazine. Incubation with PE STBEVs resulted in significantly stronger contractions during cumulative concentrations of Ang II compared to normal STBEVs (p = 0.027), with a significantly (p = 0.008) higher E_max_ of 97.3 ± 10.1% for PE vesicles compared to 76.5 ± 10.7% for normal vesicles (Fig. 1A and Table 3). However, the potency remained similar at a pEC_50_ of 10.5 and 10.1 log M, respectively (Table 3). Moreover, normal STBEVs reduced constriction to Ang II compared to vehicle only (p = 0.008) with an E_max_ of 87.6 ± 12.6% (Fig. 1A and Table 3). Endothelial function can affect vascular contractility, but there was no correlation between E_max_ for Ang II or ET-1 and the dilatory response to carbamylcholine chloride (Supplementary Figure S2). Pre-incubation with the vesicular uptake-blocker chlorpromazine significantly inhibited the contractile effect of Ang II for both PE and normal STBEVs (PE vs PE + chlorpromazine p = 0.008, and normal vs normal + chlorpromazine p = 0.004) with lower E_max_ values (36.0 ± 8.0% for PE vesicles and 48.6 ± 17.1% for normal vesicles) and lower pEC_50_ (8.9 and 8.7, respectively) (Fig. 1A and Table 3). The addition of the AT1R-blocker candesartan significantly inhibited the contraction response (p = 0.008) (Supplementary Figure S3A). Incubation with PE or normal STBEVs affected the ET-1 mediated contraction to a lesser extent, where incubation with normal STBEVs resulted in stronger contraction compared to PE STBEVs (p = 0.008), and with E_max_ of 153.5 ± 15.6% for PE and 170.1 ± 21.2% for normal STBEVs, compared to vehicle only with E_max_ of 147.3 ± 13.9 (Fig. 1B and Table 3). The pEC_50_ remained similar (Table 3). Pre-incubation with the vesicular uptake-blocker chlorpromazine inhibited the contraction by ET-1 to a lesser extent than it did for Ang II (PE vs PE + chlorpromazine p = 0.055, and normal vs normal + chlorpromazine p = 0.004), and the effect was only observed at the lower concentrations of ET-1 (Fig. 1B). The addition of 1 µM ET_A_-receptor antagonist FR139317 had some but not significant effect on the contraction response induced by ET-1 (Supplementary Figure S3B).

**Table 3:** Contraction of human subcutaneous arteries. Data presented as mean ± SEM.

	Angiotensin II		Endothelin-1	
	E_max_ %	pEC_50_ (log M)	E_max_ %	pEC_50_ (log M)
Normal STBEV	76.5 ± 10.7	10.1	170.1 ± 21.2	9.5
PE STBEV	97.3 ± 10.1**	10.5	153.5 ± 15.6	9.6
Normal STBEV + chlorpromazine	48.6 ± 17.1	8.7	147.8 ± 32.9	9.2
PE STBEV + chlorpromazine	36.0 ± 8.0	8.9	156.2 ± 25.4	8.6
Vessels without STBEV (vehicle)	87.6 ± 12.6	10.8	147.3 ± 13.9	9.5

PE = preeclampsia; STBEV = syncytiotrophoblast-derived extracellular vesicles; E_max_ = maximum contractile response; pEC_50_ = negative logarithm of the half maximum effective concentration. Non-parametric Wilcoxon’s matched-pairs signed-ranks test: **p < 0.01.

### Transmission electron microscopy analysis of STBEV-exposed vessel morphology

**Figure 2:**
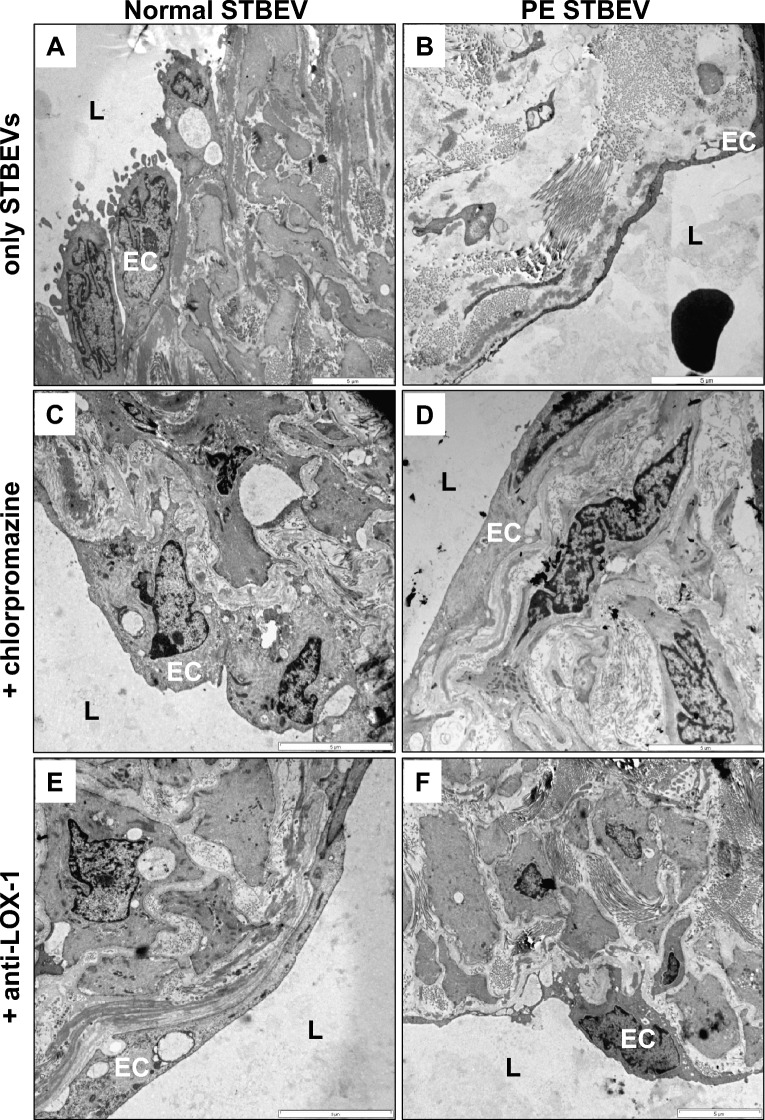
TEM analysis of tissue morphology in STBEV exposed human arterial vessels. Transmission electron microscopy analysis of human arterial vessels exposed in vitro to placenta-derived STBEVs. (**A**) Exposure to normal STBEVs. (**B**) Exposure to PE STBEVs. (**C**) Exposure to normal STBEVs in combination with chlorpromazine. (**D**) Exposure to PE STBEVs in combination with chlorpromazine. (**E**) Exposure to normal STBEVs in combination with anti-LOX-1 antibody. (**F**) Exposure to PE STBEVs in combination with anti-LOX-1 antibody. Scale bar = 5 µm. EC = endothelial cell; L = arterial vessel lumen.

Transmission electron microscopy analysis of human arterial vessels exposed to normal STBEVs showed dark dense intact morphology (Fig. 2A) as seen in the control vessels not exposed to STBEVs (Supplementary Figure S4). Thick endothelial cells were anchored to the basement membrane, next to dense tunica intima and tunica media with compact extracellular matrix (ECM) structures surrounding SMCs (Fig. 2A). In contrast, vessels exposed to PE STBEVs showed a light loose morphology, with thin and extended endothelial cells, a loose thicker tunica intima, a tunica media with disorganized ECM showing separated and fragmented collagen fibers as well as detached vascular SMCs (Fig. 2B). Addition of the clathrin-mediated uptake blocker chlorpromazine together with PE STBEVs protected the ultra-morphology and prevented disruption of ECM as observed in a darker dense organized tunica media and normal endothelial cells (Fig. 2D). LOX-1 is expressed at low levels in vessels from normal pregnancies^21^ but even so the addition of an anti-LOX-1 antibody together with PE STBEVs preserved tissue integrity to a certain extent, preventing disorganization of tunica media and ECM albeit with thinner but intact endothelial cells (Fig. 2F) compared to normal STBEVs (Fig. 2A). Exposure to normal STBEVs in combination with chlorpromazine (Fig. 2C) or an anti-LOX-1 antibody (Fig. 2E) showed normal morphology comparable to the normal STBEV exposure (Fig. 2A) or control vessels not exposed to STBEVs (Supplementary Figure S4).

### Visualizing STBEV uptake and transport by endothelial cells

**Figure 3:**
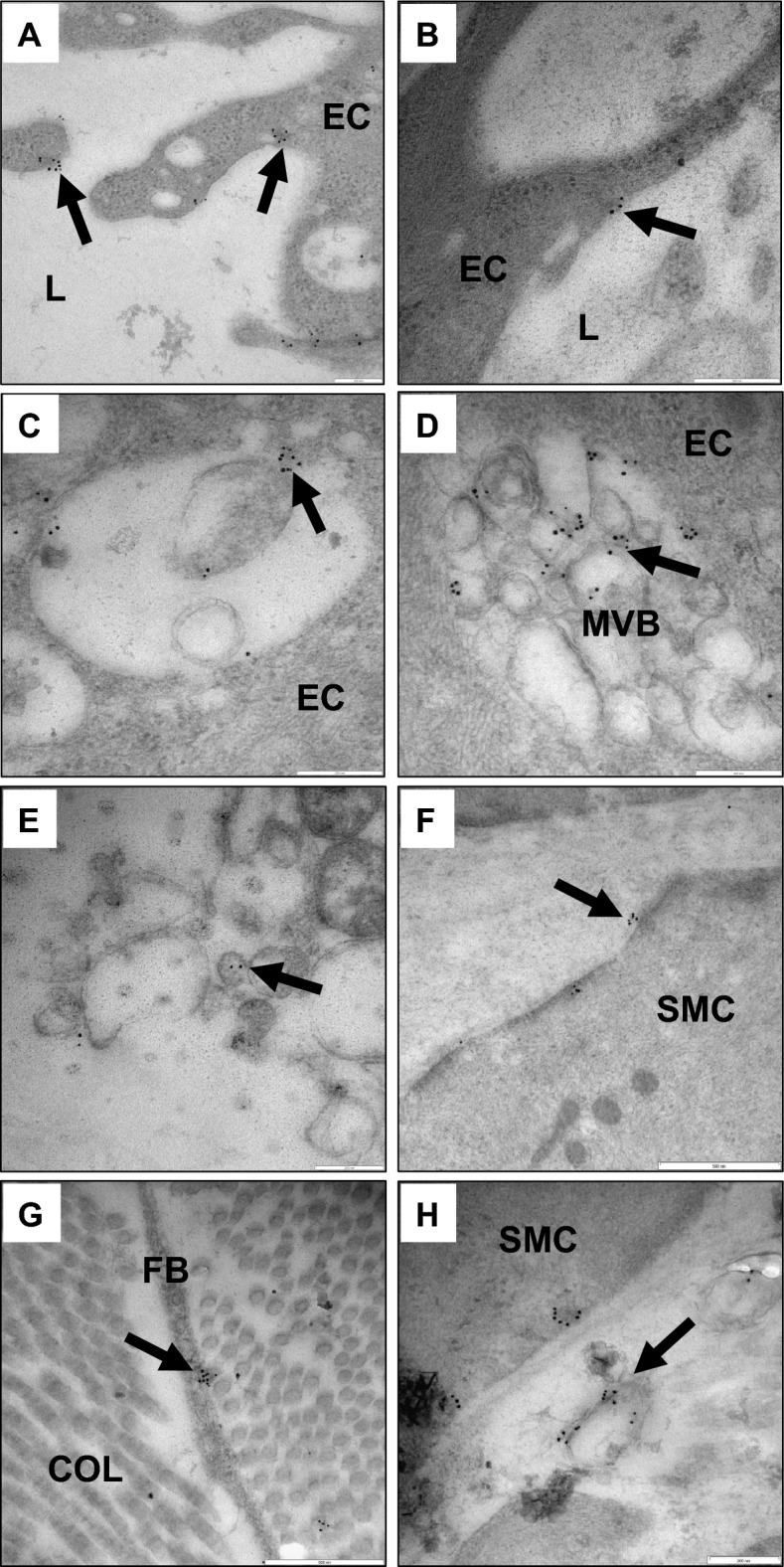
STBEVs are taken up by arterial endothelial cells. Immunoelectron microscopy analysis using gold labeled anti-PLAP antibodies on human arterial vessels exposed in vitro to placenta derived STBEVs. (**A**) Exposure to normal STBEVs. Black arrows indicate PLAP-positive STBEVs attaching to the endothelial cell plasma membrane. (**B**) Exposure to PE STBEVs. PLAP-positive STBEV attaches to the endothelial cell plasma membrane. (**C**) Exposure to normal STBEVs. PLAP-positive STBEV inside an endosome in the endothelial cell. (**D**) Exposure to normal STBEVs. Multivesicular body containing multiple PLAP-positive STBEVs. (**E**) Exposure to PE STBEVs. PLAP-positive STBEV trapped inside an endosome among cell debris. (**F**) PLAP detected on SMC inside the tunica media. Scale bar = 500 nm. (**G**) PLAP detected on fibroblast cell among collagens. Scale bar = 500 nm. (**H**) Endosome-structures carrying PLAP-positive STBEVs close to SMCs. Scale bar = 200 nm. EC = endothelial cell; L = arterial vessel lumen; MVB = multivesicular body; SMC = smooth muscle cell.

**Figure 4:**
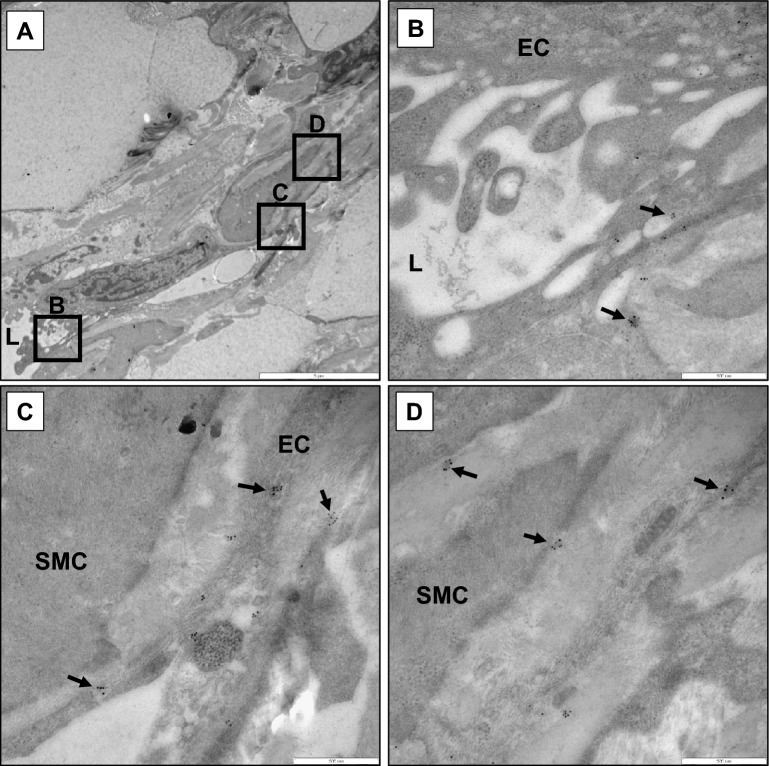
STBEV uptake and transport to smooth muscle cells. Immunoelectron microscopy analysis using gold labeled anti-PLAP antibody staining on human arterial vessels exposed in vitro to normal STBEVs. (**A**) Overview of the vessel wall with the vascular lumen and endothelial lining at the bottom left. Two neighboring endothelial cells were seen protruding into the vessel wall. Squares represent magnifications displayed in sections B-D. Scale bar = 5 µm. (**B–D**) Magnification of areas from section A, showing PLAP-positive STBEVs transported along endothelial plasma membrane protrusions towards SMCs. Scale bar = 500 nm. EC = endothelial cell; L = arterial vessel lumen; SMC = smooth muscle cell.

Placental alkaline phosphatase (PLAP), expressed on placenta-derived extracellular vesicles, was used to visualize STBEVs by immunoelectron microscopy analysis using gold-labeled antibodies. These analyses showed PLAP-positive normal (Fig. 3A) and PE STBEVs (Fig. 3B) attached to the plasma membrane of the endothelial cells. In addition, PLAP-positive STBEVs were detected inside endosomes in the endothelial cells, as well as attached to the endosome membrane (Fig. 3C). Normal STBEVs were found in both early and late endosomes, also called multivesicular bodies, with multiple STBEVs attached (Fig. 3D). In vessels exposed to PE STBEVs, the interrupted ECM and changed endothelial morphology made it difficult to detect STBEVs in conjunction with any defined structures beyond the endothelial plasma membrane and early endosomes (Fig. 3E). In vessels exposed to normal STBEVs and focusing on intact tissue closest to the endothelial layer, the STBEVs could be detected inside the vessel wall attached to SMCs (Fig. 3F) and fibroblasts (Fig. 3G). In these areas, endosomes, or parts of multivesicular bodies, were found carrying multiple STBEVs (Fig. 3H). Also, in these vessels, STBEVs were visualized being transported along endothelial plasma membrane protrusions that extended inwards into the vessel wall next to a SMC (Fig. 4). In control vessels not exposed to STBEVs (Supplementary Figure S5B), PLAP staining demonstrated naturally occurring STBEVs in pregnancy that could, as rare occurrences, be detected in tunica media or intima but not along the endothelial plasma membrane (Supplementary Figure S5C).

### Blocking STBEV uptake by endothelial cells

**Figure 5:**
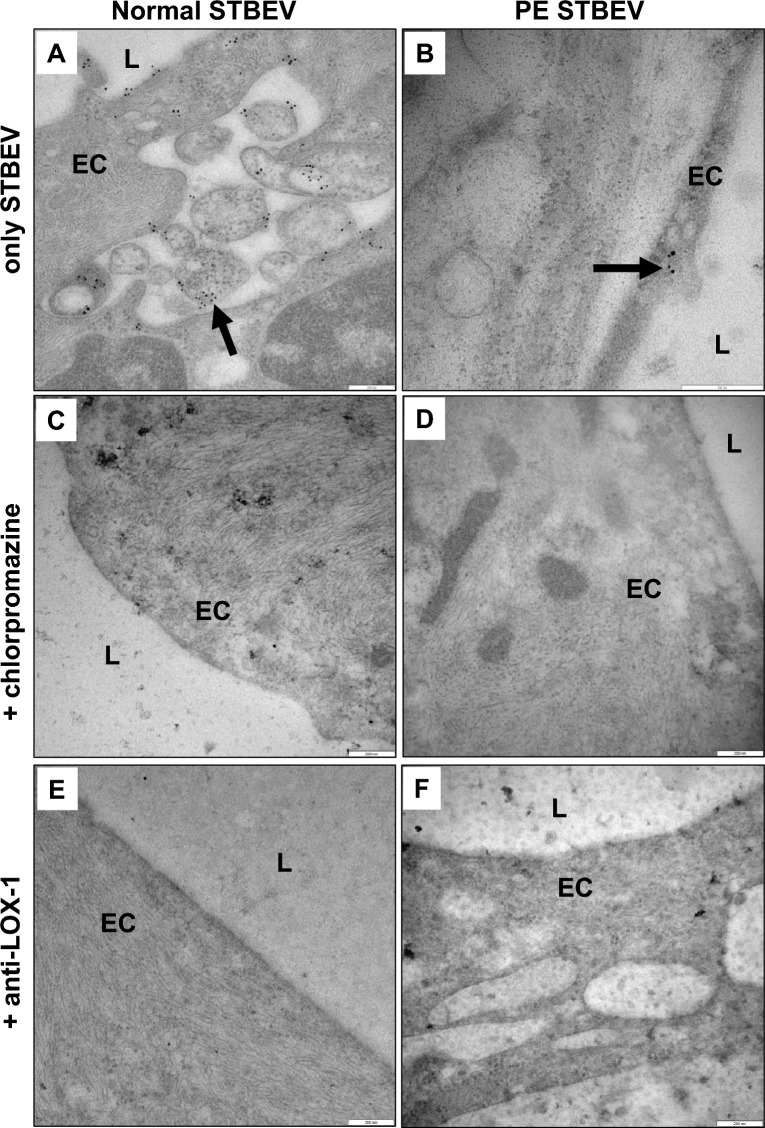
Blocking STBEV uptake in exposed human arterial vessels. Immunoelectron microscopy analysis using gold labeled anti-PLAP antibodies on human arterial vessels exposed in vitro to placenta-derived normal (**A**, **C**, **E**) or PE (**B**, **D**, **F**) STBEVs. (**A**) Exposure to normal STBEVs. Black arrows indicate PLAP-positive STBEVs. (**B**) Exposure to PE STBEVs. Black arrow indicates PLAP-positive STBEV. (**C**) Exposure to normal STBEVs in combination with chlorpromazine. (**D**) Exposure to PE STBEVs in combination with chlorpromazine. (**E**) Exposure to normal STBEVs in combination with anti-LOX-1 antibody. (**F**) Exposure to PE STBEVs in combination with anti-LOX-1 antibody. Scale bar = 200 nm. EC = endothelial cell; L = arterial vessel lumen.

Vessels were exposed to normal or PE STBEVs in vitro in combination with the uptake blocker chlorpromazine or a specific anti-LOX-1 blocking antibody. By using immunoelectron microscopy analysis, we showed that chlorpromazine treatment prevented PLAP-positive staining on the endothelial plasma membrane and inside endosomes in the endothelial cells exposed to normal (Fig. 5C) or PE STBEVs (Fig. 5D), as well as on SMCs (Supplementary Figure S6A), compared to no blocking (Fig. 5A, B). A similar pattern was seen when combining normal or PE STBEV exposure with the anti-LOX-1 antibody, no STBEVs were attached to the plasma membrane or inside endosomes (Fig. 5E, F) as well as on SMCs (Supplementary Figure S6B). Despite the reported low levels of LOX-1 in vessels from normal pregnancies^21^ we could detect LOX-1 with immunoelectron microscopy in normal subcutaneous arteries not exposed to vesicles (Supplementary Figure S7A) on endothelial plasma membrane. It was also present inside endosomes in endothelial cells and inside SMCs, most often two or more receptors together (Supplementary Figure S7B-C).

## Discussion

We investigated the vascular and morphological effects of normal and PE STBEVs on human subcutaneous arteries in vitro. The vesicles were obtained from previously ex vivo perfused normal or PE placentas. To the best of our knowledge, this is the first study to investigate the effects of human placenta-derived normal and PE STBEVs on pregnant human arteries. Our results showed that PE STBEVs caused significantly stronger AT1R-mediated contractions and more extended structural damage to the vessel wall compared to normal STBEVs, that in contrast caused a reduced contraction in presence of Ang II. Additionally, this study provides preliminary evidence that the negative effects of PE STBEVs on cellular and morphological structure of the arteries can be prevented by pharmacological blocking of vesicular uptake, in this case by chlorpromazine or by a specific anti-LOX-1 receptor blocking antibody. The results demonstrated the possible roles played by clathrin-mediated endocytosis and the LOX-1 receptor in mediating uptake and effects of STBEVs on maternal subcutaneous arteries.

Elevated plasma levels of STBEVs in PE are believed to relate to different stressors affecting the placenta, contributing to increased shedding, and altered cargo^15,17^. Our results showed that PE STBEVs induced stronger Ang II-mediated contractions, via the AT1R subtype in human subcutaneous arteries similar to the increased and persistent peripheral resistance^41^ and vascular sensitivity to Ang II^19^ observed in PE. In contrast, normal STBEVs reduced contraction in the presence of Ang II compared to the untreated artery (vehicle, buffer only) which is in accordance with the adaptive reduction of the peripheral resistance in normal pregnancies at mid pregnancy^41^. Similar experiments in normal pregnant mice have shown that vasodilation by normal STBEVs is due to a decrease in Ang II responsiveness, suggested to be mediated by decreased levels of AT1R^42^. Interestingly, these vascular adaptations were absent in LOX-1 overexpressing mice^42^, that mimic PE with same elevated levels of LOX-1 in arterial vessels^21^. We suggest that normal STBEVs facilitate the internalization of AT1R via normal low levels of LOX-1 receptor and a β-arrestin pathway involving clathrin-mediated endocytosis, to attenuate Ang II signaling as a desensitization process in normal pregnancy adaptation. With increased levels of STBEVs^43^ and increased LOX-1 expression in maternal vasculature in PE^21^, or with increased levels of oxLDL seen in other pathologies such as atherosclerosis^44^, the internalization process may instead become pathological.

Compared to Ang II, we found a smaller difference between normal and PE STBEV in ET-1-mediated vascular contraction. This is in line with Simsek et al.^45^, who described a significantly higher ET-1 mRNA expression in PE placentas, but no difference in contraction using PE or control umbilical arteries. In both in vitro and in vivo models, Ang II treatment has been shown to drive ET-1 expression, but it is most important as the initial pressor of Ang II-induced blood pressure regulation^46^. Considering that ET-1 expression is also regulated by hypoxia^47,48^, our results suggest a central role for PE STBEV regulation of Ang II-induced vasocontraction which, consequentially or in combination with local hypoxia, leads to increased ET-1 expression in PE.

We have previously shown that STBEV internalization by endothelial cells^32^ involves clathrin-mediated endocytosis^14^. In addition, STBEVs mediate their effects on endothelial cells via the LOX-1 receptor^42^, which in turn interacts with the AT1R causing internalization of the two receptors via clathrin-mediated endocytosis^24^. By blocking STBEV uptake, we showed that both clathrin-mediated endocytosis and the LOX-1 receptor play a role in endothelial vesicle uptake. We visualized the STBEVs attaching to the endothelial plasma membrane and localizing inside the endothelial cells in early and late endosomes. Our analysis also detected STBEVs attached to SMCs and fibroblasts inside the vascular wall. Endosomes or parts of multivesicular bodies carrying multiple STBEVs were present in the tunica media, suggesting transport of STBEVs across the endothelial cells, to target the SMCs. Multivesicular bodies have been shown to fuse with the endothelial plasma membrane and release its internal vesicles as exosomes^49^. Another possibility for transport of intact STBEVs could be transcytosis, which is a transcellular process that allows material to enter a cell on one side and exit via the opposite side^50^. In atherosclerosis, transcytosis is used by vascular endothelial cells to transport LDL and even oxLDL across the cell to be released into the arterial wall^51^. We also noted that STBEVs seemed to be transported along endothelial cell plasma membrane protrusions, reaching deeper into the vessel wall towards the SMC layer underneath. Blocking STBEV uptake by chlorpromazine or an anti-LOX-1 antibody seems to abolish this STBEV transport, with no delivery to SMCs. This supports the idea that a natural route for placenta-derived STBEVs is via uptake by endothelial cells, to contribute to vascular adaptations during pregnancy. Studies on rat arteries have shown that a LOX-1 blocking antibody in myograph experiments restored the vasodilation response to methylcholine^25^, suggesting a blocked internalization of the STBEV-LOX-1-AT1R complex.

Chlorpromazine blocked the STBEV uptake and prevented the contractile response and tissue degradation by PE STBEVs. The TEM analysis indicated that chlorpromazine prevented attachment of STBEVs to the endothelial plasma membrane. Chlorpromazine is known to block Ang II mediated contractions in rat and guinea pig aorta^52^, remove clathrin from the plasma membrane, as well as inhibiting receptor recycling^53^. Our study suggests that the effect of chlorpromazine is primarily related to the Ang II-mediated contraction and not to ET-1, that mainly involves caveolae and intracellular pathways associated with intracellular calcium^54^ and is less affected by chlorpromazine^28^. Future studies are required to explore the therapeutic potential of blocking STBEV uptake, to reduce the permanent damage seen on the maternal vasculature in PE.

The TEM analysis showed damage to the vascular endothelial cells exposed to the PE but not to normal STBEVs. Other in vitro studies have shown that PE STBEVs suppress proliferation and survival of endothelial cells but have no effect on non-endothelial cell lines^55^. This points to the specific interaction between STBEVs and endothelial cells and is in line with general cell injury in the maternal vascular bed, a hallmark of PE. This is supported by increased numbers of detached circulating endothelial cells in PE, as well as increased plasma levels of markers for endothelial activation, dysfunction, and damage^56^.

Arterial ECM is mainly formed of collagen, fibronectin and elastin along with a wide range of other proteins^57^. The ECM is an active and dynamic structure that takes part in the regulation of vascular tonus^57^. Damage to the ECM contributes to vascular diseases, and results in release of degradation products with pathophysiological functions^57^. In the current study, PE STBEVs had a disruptive effect on the basement membrane underneath the endothelial cells, as well as on the tunica intima and tunica media. Disruption and fragmentation of the ECM led to detachment of vascular SMCs. Elevated maternal plasma levels of degradation products reported in the third trimester of PE pregnancies, indicates degradation of basement membranes and ECM^58,59^. Damage to vascular elastin and collagen can lead to excess collagen expression and deposition, which can increase vascular stiffness and alter endothelial cell behavior^57^. This can contribute to the increased arterial stiffness and long-term cardiovascular effects seen after PE. Considering the therapeutic opportunity with blocking vesicular uptake, future studies need to focus on the possibility to protect the maternal vasculature from the effects of PE STBEVs.

A weakness of our study is that the LOX-1 blocking antibody was not available to us at the time of the myograph experiments. However, supporting our study are previous myography experiments showing that an anti-LOX-1 antibody significantly reduces the effects of STBEVs on rat arteries^25^. Another weakness is that the available early-onset and late-onset PE STBEVs were not sufficient to allow for comparison between the two separate subsets to shed light on the potential pathological differences between these forms of PE. The placental dual ex-vivo perfusion technique is cumbersome and presents many challenges and therefore not established in many laboratories. In addition, PE placentas present even more of a challenge due to pathological damage and leakiness. However, our study indicates the importance of future experiments to elucidate any differences in effect on maternal vessels between early- and late-onset STBEVs as well as any potential sexual dimorphism. A strength of our study is that it demonstrates the impact of human placenta-derived STBEVs on human vessels from normal pregnancies instead of using animal models or animal derived arteries.

Regarding the dose of STBEVs used in our in vitro experiments (40 µg/ml), it is lower than in similar in vitro myograph experiments using 200 µg/ml of human normal or PE STBEVs on animal arteries^42,60^ or human normal STBEVs on human subcutaneous arteries^61^. In our previous in vitro blocking experiments on cultured cells, we used 10 µg/ml STBEVs with 1 h incubation, which was chosen after a dose response experiment^14^. The dose in this study was in between these two and the incubation time shorter than similar myograph experiments to ensure the viability of the exposed vessels. We have previously shown that placenta-derived STBEVs are taken up by human endothelial cells within 30 min of exposure^32^. In general, in vitro experiments use a higher dose of STBEVs compared to reported in vivo doses determined in maternal circulation^15^.

In conclusion, PE-derived STBEVs caused significantly stronger Ang II-mediated vasoconstriction and extended structural damage to human subcutaneous arteries in vitro, compared to STBEVs from normal placentas that in contrast reduced contraction without any tissue damage. We suggest that altered cargo of PE STBEVs contributes to maternal vascular dysfunction and endothelial damage. The observed structural damage may eventually lead to the stiffening of the vascular bed seen after PE, which may underlie the increased risk of CVD. This study provides preliminary evidence that the negative effects caused by PE STBEVs could be reduced by pharmacological blocking of clathrin-mediated vesicle uptake, in this case by chlorpromazine or by blocking the LOX-1 receptor. Future studies will explore the therapeutic potential of blocking STBEV uptake in PE, to reduce the permanent damage caused on the vasculature thereby potentially reducing the risk of CVD in these women.

### Supplementary Information

Supplementary Information.

## Data Availability

L.E. and L.O. had full access to all the data in the study and takes responsibility for its integrity and the data analysis. All data is included within the main text or in Supplementary information.
