# Supplementary material for: Preliminary evidence that blocking the uptake of placenta-derived preeclamptic extracellular vesicles protects the vascular endothelium and prevents vasoconstriction
Source: Sci Rep. 2023 Oct 27;13:18425. doi: 10.1038/s41598-023-45830-9 (PMC10611745; doi:10.1038/s41598-023-45830-9)
Supplement: Supplementary file 1 — Supplementary Information. [file 41598_2023_45830_MOESM1_ESM.pdf]

**Supplementary information**

# Pharmacological blocking of uptake of placenta-derived preeclamptic extracellular vesicles protects the vascular endothelium and prevents vasoconstriction

Lena Erlandsson[1#*], Lena Ohlsson[2#], Zahra Masoumi[1], Mimmi Rehnström[1], Tina Cronqvist[1], Lars Edvinsson[2,3] and Stefan R. Hansson[1,3]

1. Division of obstetrics and gynecology, Department of Clinical Sciences Lund, Lund University, Lund, Sweden
2. Experimental vascular research, Department of Clinical Sciences Lund, Lund University, Sweden
3. Skåne University Hospital, Lund/ Malmö, Sweden

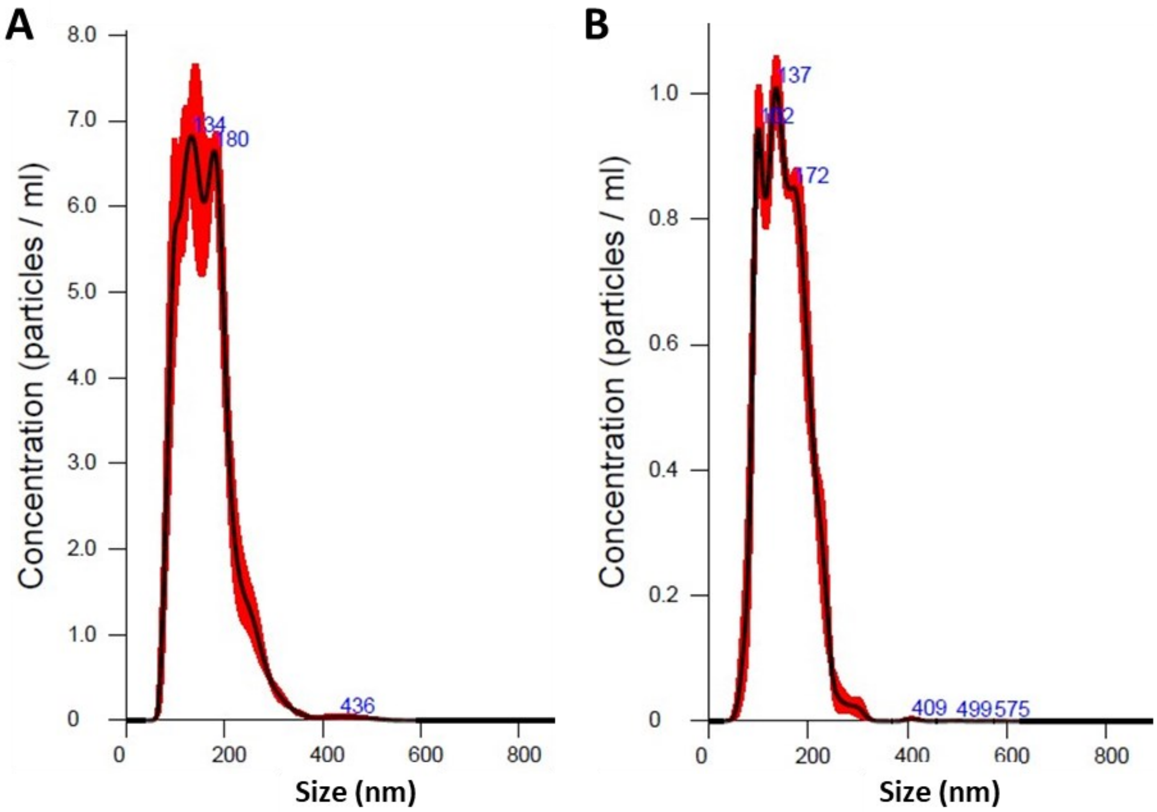


**Supplementary Figure S1. Analysis of size distribution in normal and PE STBEVs**

Representative Nanoparticle Tracking Analysis profiles showing mean of 3 consecutive runs for (A) normal and (B) PE STBEVs, to determine size distribution and particle concentration.

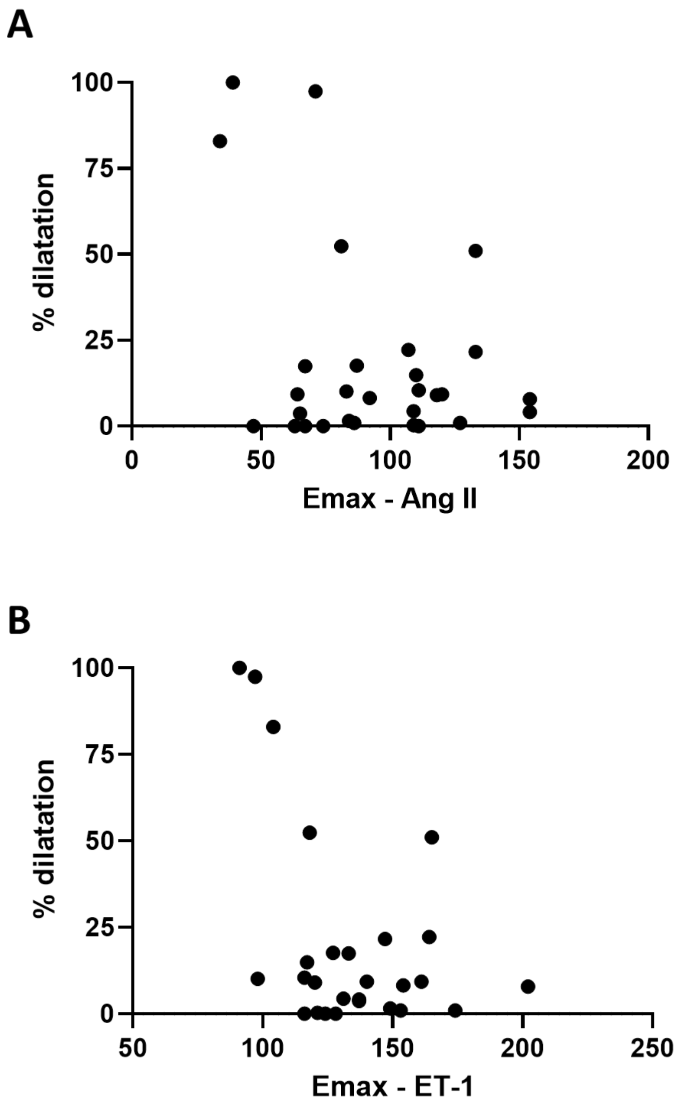


**Supplementary Figure S2. Correlation between endothelial function and $E_{max}$**

Correlation analysis using Spearman's rank correlation coefficient between endothelial function and (A) $E_{max}$ for Ang II (r = -0,017, p = 0,931, n = 29) and (B) $E_{max}$ for ET-1 (r = -0,269, p = 0,174, n = 27).

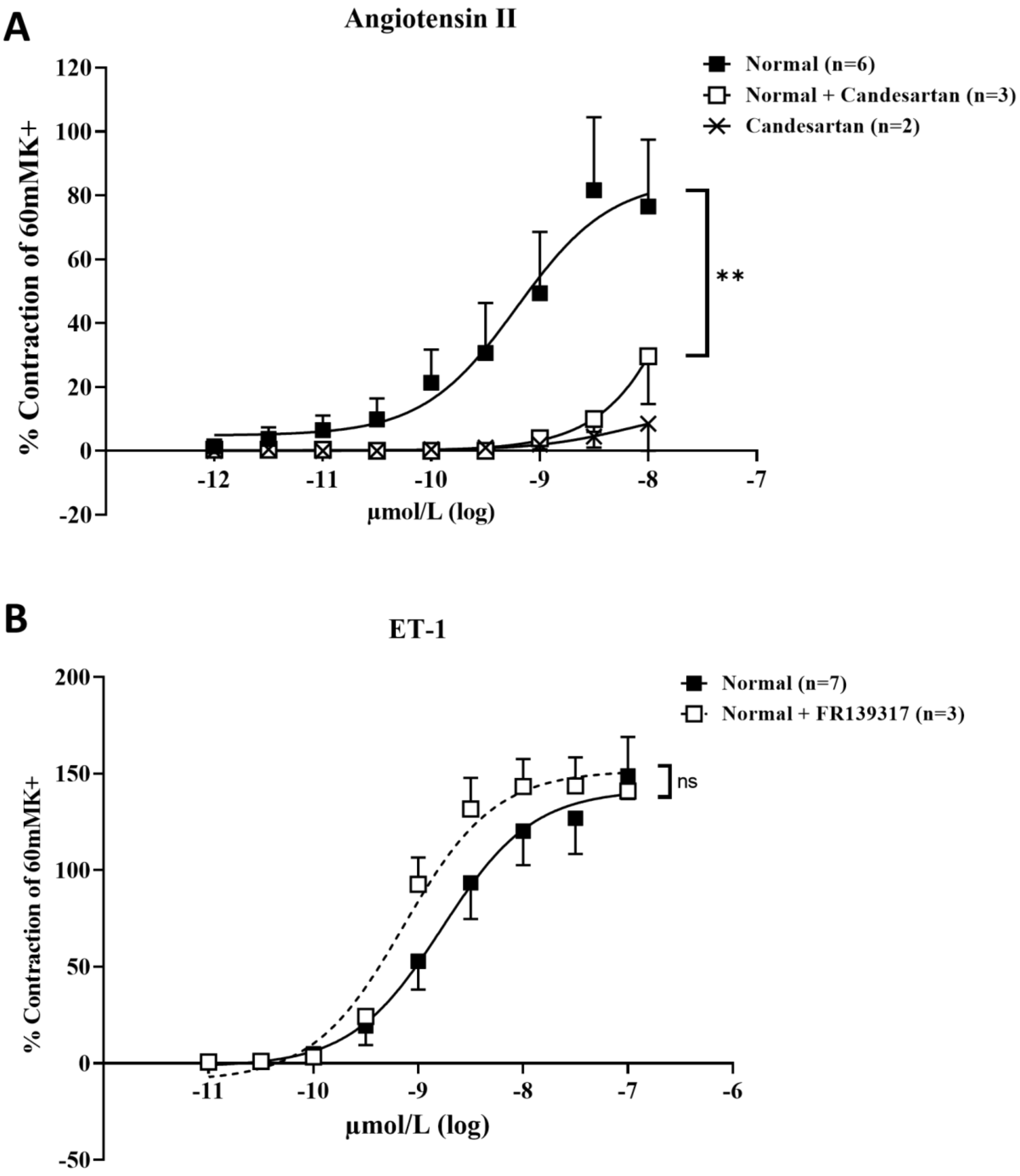


**Supplementary Figure S3. Myograph analyses of STBEV exposed mounted human arterial vessels**

Human arterial vessels mounted in a myograph and incubated with normal STBEVs. (A) Contractility response to normal STBEVs at increasing concentrations of Angiotensin II, with or without candesartan. (B) Contractility response to normal STBEVs at increasing concentrations of Endothelin-1, with or without FR139317. Data is presented as mean ± SEM for each data point. **p<0.01.

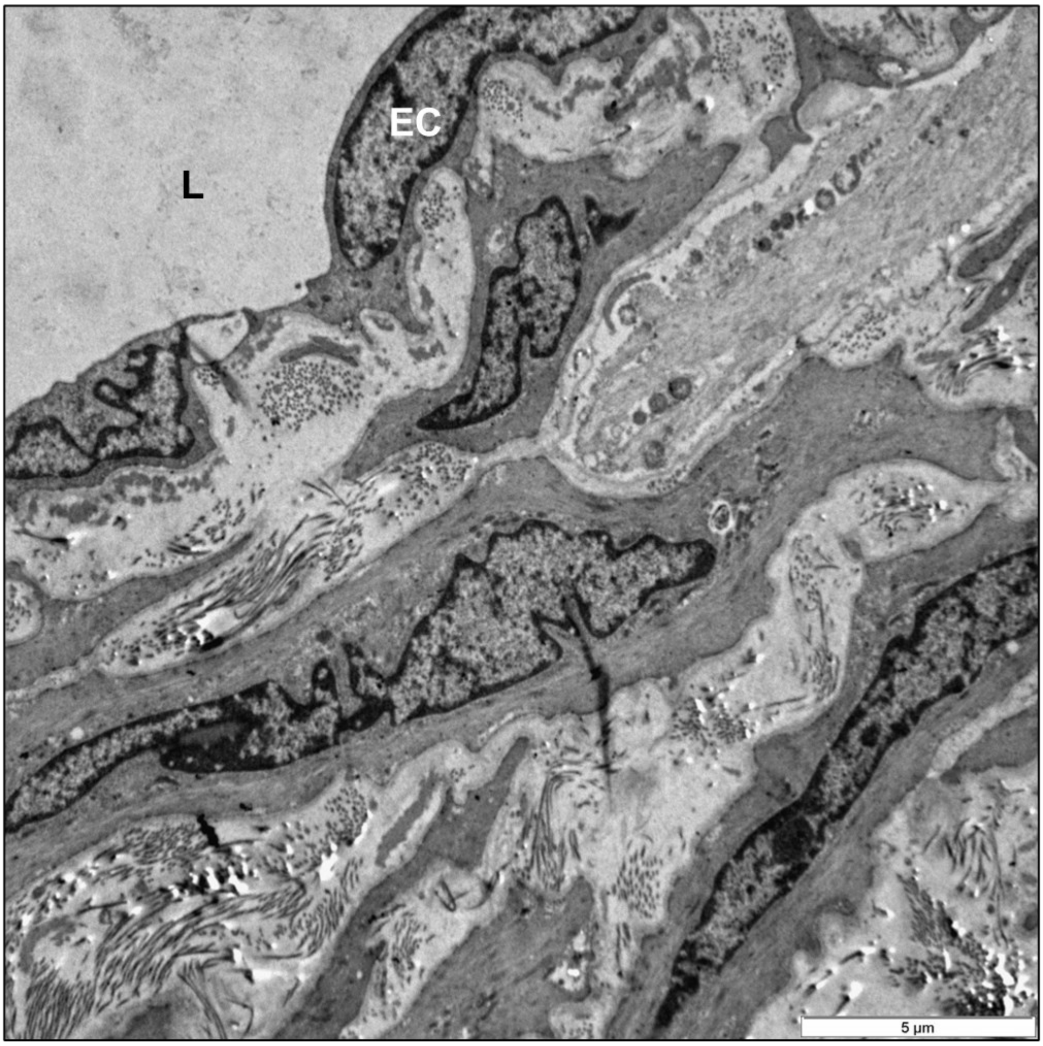


**Supplementary Figure S4. TEM analysis of STBEV exposed human arterial vessels** Control vessel not exposed to vesicles. Scale bar = 5µm. EC = endothelial cell; L = arterial vessel lumen.

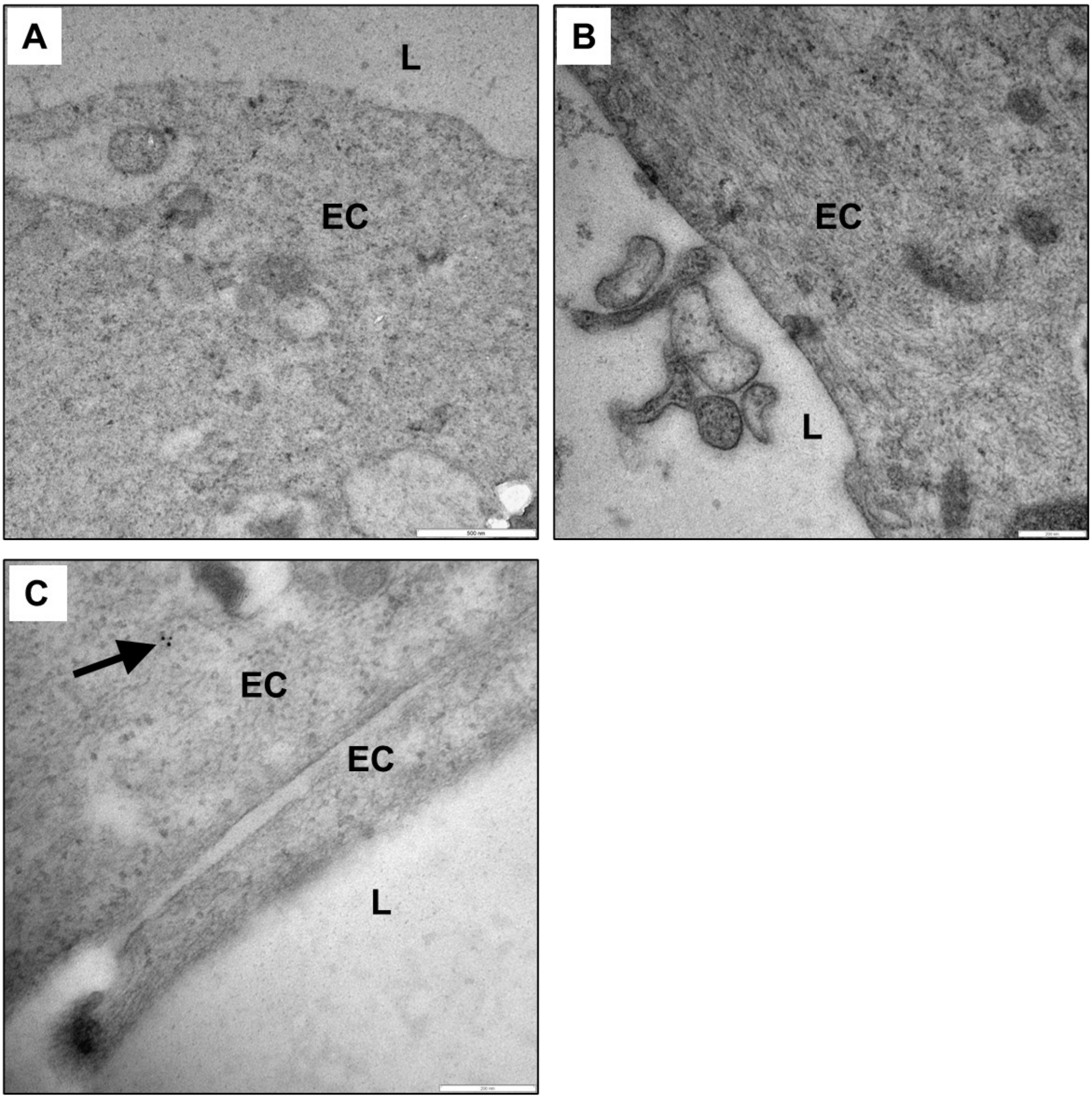


**Supplementary Figure S5. Immunoelectron analysis of STBEV exposed human arterial vessels**
Immunoelectron microscopy analysis using gold labeled anti-PLAP antibodies on human arterial vessels exposed *in vitro* to placenta derived STBEVs. (A) Negative control for immunoelectron microscopy. Scale bar = 500nm. (B) PLAP staining on control vessel not exposed to STBEVs. Scale bar = 200nm. (C) PLAP staining of control vessel not exposed to STBEVs and black arrow indicating naturally occurring PLAP-positive vesicles from normal pregnancy. Scale bar = 200nm. EC = endothelial cell; L = arterial vessel lumen.

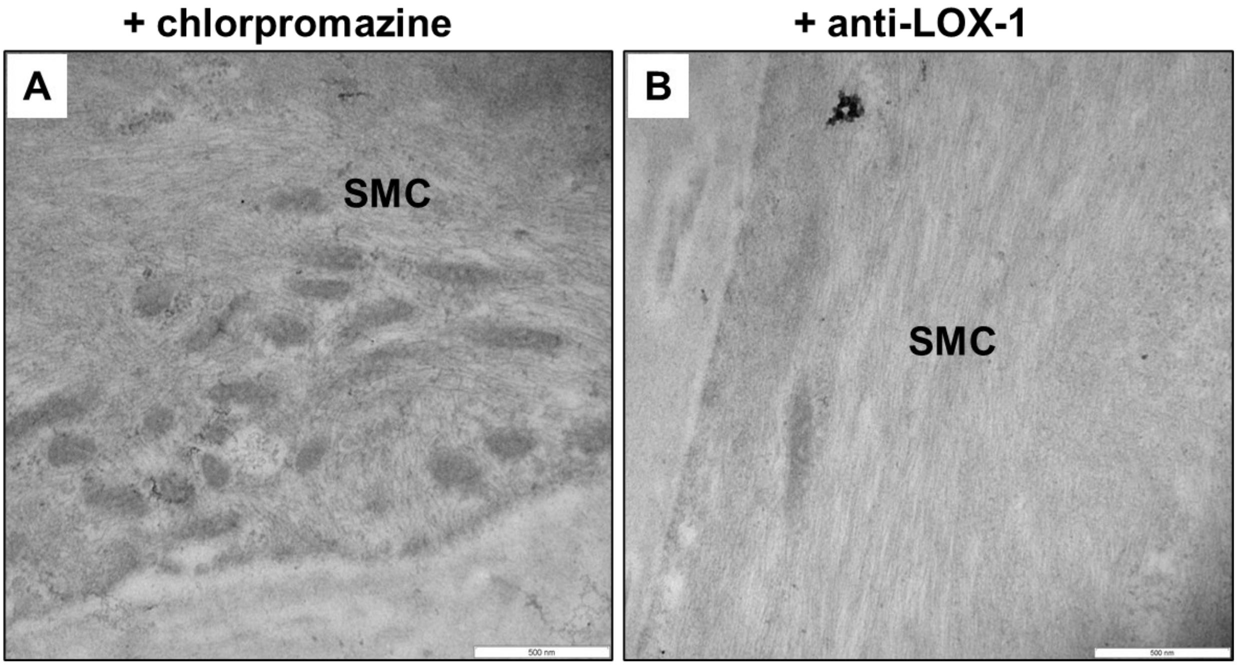


**Supplementary Figure S6. Immunoelectron analysis of STBEV exposed human arterial vessels**
Immunoelectron microscopy analysis using gold labeled anti-PLAP antibodies on human arterial vessels exposed *in vitro* to placenta derived STBEVs. (A) PLAP staining of vessel exposed to normal STBEVs and chlorpromazine. (B) PLAP staining of vessel exposed to normal STBEVs and anti-LOX-1 antibody. Scale bar = 500nm. SMC = smooth muscle cell.

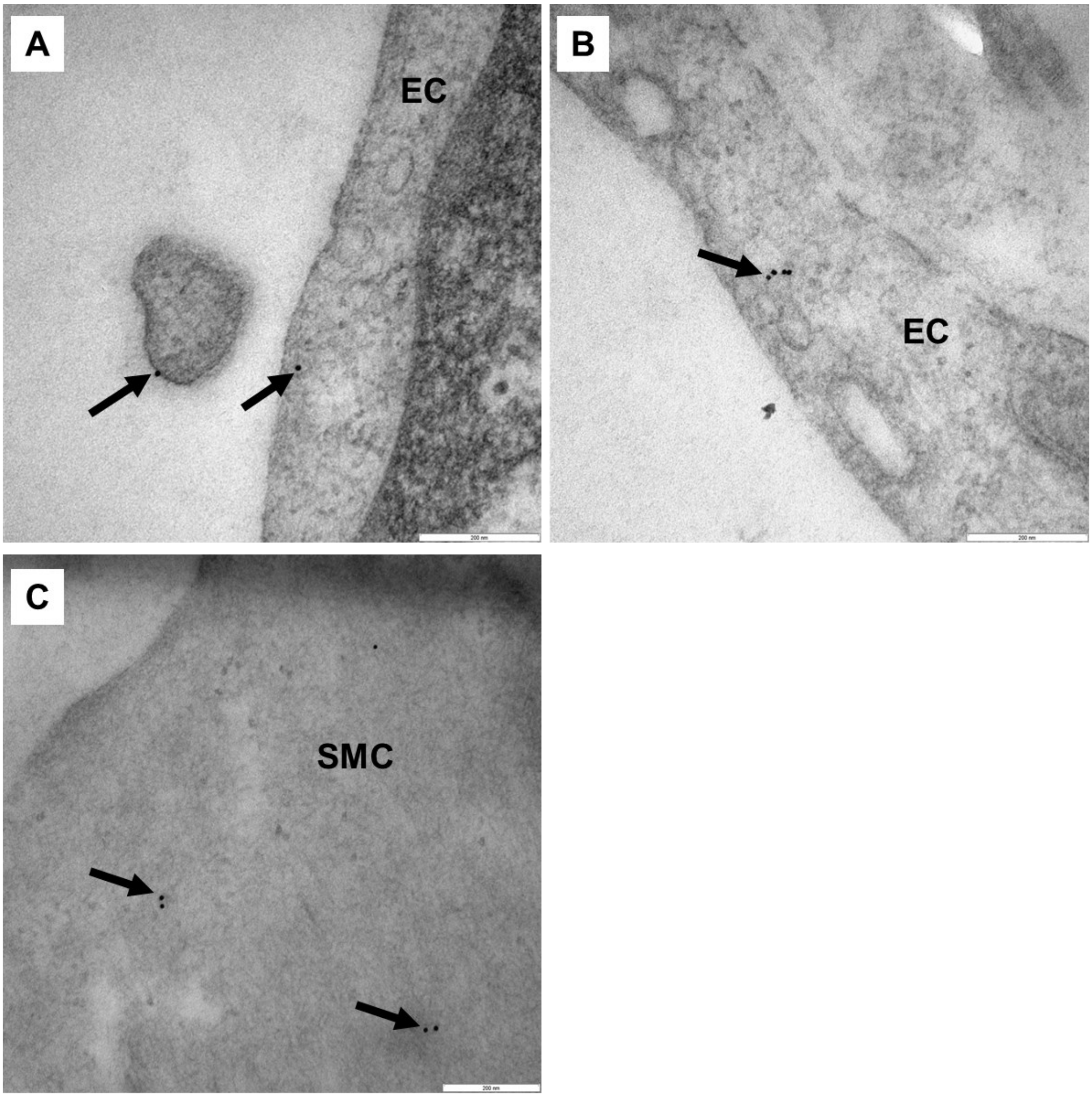


**Supplementary Figure S7. Immunoelectron analysis of human arterial vessels**
Immunoelectron microscopy analysis using gold labeled anti-LOX-1 antibodies on human arterial vessels not exposed to STBEVs. Black arrows indicates LOX-1 on (A) endothelial plasma membrane, (B) on an endosome inside an endothelial cell, and (C) inside a smooth muscle cell. Scale bar = 200nm. EC = endothelial cell; SMC = smooth muscle cell.
